# Implementation of rural provider-to-provider telehealth in country Western Australia: a retrospective observational analysis via the RE-AIM framework

**DOI:** 10.1186/s12913-025-12335-2

**Published:** 2025-01-31

**Authors:** Kaylie Toll, Suzanne Robinson, Stephen Andrew, Aled Williams, Justin Yeung, Richard Varhol, Joanna C. Moullin

**Affiliations:** 1 School of Population Health, Curtin University, GPO Box U1987, Bentley, Perth, WA 6845 Australia; 2 enAble Institute, Curtin University, Perth, WA Australia; 3 WA Country Health Service, Perth, WA Australia; 4 Deakin Health Economics, Institute for Health Transformation, School of Health and Social Development, Faculty of Health, Deakin University, Melbourne, VIC Australia

**Keywords:** Telehealth, Virtual care, Emergency care, Implementation science, Observational analysis

## Abstract

**Background:**

Rural provider-to-provider telehealth is growing globally. It is used to both facilitate equitable access to specialist healthcare services for those living in rural and remote areas and provide support to place-based providers. There is limited research on the implementation of these services, especially in an emergency or inpatient hospital setting. The Western Australia Country Health Service (WACHS) Command Centre is one such example. First implemented in 2012, the Command Centre services a geographical area covering 2.55 million square kilometres, a population of approximately 550,000, and provides five clinical streams including Emergency, Mental Health Emergency, Midwifery and Obstetrics Emergency, Inpatient, and Palliative Care Afterhours Telehealth Services.

**Objectives:**

This study aimed to evaluate the implementation and access of rural provider-to-provider telehealth in country Western Australia, for the years 2012 to 2023.

**Methods:**

A retrospective observational analysis was conducted of all patient contacts managed by clinical telehealth streams of the Command Centre, between 31 August 2012 and 31 December 2023. Utilising descriptive statistics, analyses was informed by the expanded Reach, Effectiveness, Adoption, Implementation, Maintenance (RE-AIM) Framework and Implementation Outcomes Framework (IOF) definitions.

**Results:**

Over the near 12-year period, a total of 215,965 service contacts were analysed from the five Command Centre clinical streams. There was large variation in the reach and adoption of services across regions, sites, and health facility types, however service scope and activity has increased steadily over time (maintenance). 95 of the 103 WACHS-managed sites had utilised Command Centre clinical telehealth services. The Command Centre has seen an increase in the proportion of clinical telehealth services provided to the most disadvantaged populations, demonstrating improved equity of access (effectiveness) over time.

**Conclusion:**

There is a steady expansion in the availability of provider-to-provider clinical telehealth services delivered by the WACHS Command Centre across country WA, but with wide variability of usage depending on region, site, and health facility type. The results of this study show there is a need to understand the contextual factors influencing the adoption, implementation, and sustainability of the service.

**Supplementary Information:**

The online version contains supplementary material available at 10.1186/s12913-025-12335-2.

## Background

Rural and remote areas are consistently associated with a lack of access to healthcare and health workforce shortages [1]. These inequalities can result in poorer health outcomes for those living in these areas, including higher rates of hospitalisation, morbidity, mortality, and burden of disease [1]. Geographical distance and economies of scale continue to be an ongoing challenge for health systems deciding to deliver people to services or services to people [2]. Enhancing healthcare utilisation is achieved by increasing access to services, a key sustainability pillar for healthcare echoed globally [3, 4]. Healthcare enabled by technology has been found to improve the accessibility to healthcare by increasing the coverage of specialist services and delivering care closer to home [3, 5].

Rural provider-to-provider telehealth (RPPT) is one such model of care, used in emergency, inpatient, and outpatient care and defined as a collaboration between rural and virtual providers in the delivery of healthcare to those living in rural and remote areas [6]. This model of care reflects and expands on the American College of Emergency Physicians (ACEP) definition of emergency telehealth, “remotely caring for acute illness, injury and exacerbations of chronic diseases, including the initial evaluation, diagnosis, treatment, prevention, coordination of care, disposition and public-health impact of any patient requiring expeditious care, irrespective of any prior relationship” [7]. Whilst the ACEP definition covers multiple environments including hospitals and pre-hospital settings, maritime and military settings, and direct to consumer services; RPPT is focused specifically on the provider-to-provider connection.

Recent research has shown RPPT can bridge gaps in access to healthcare, produce results similar to face-to-face services, and increase the support provided to the rural and remote health workforce [6, 8, 9]. There are limitations to this research, including methodological weaknesses, plus a lack of clarity regarding the overall value of RPPT. By measuring and achieving a high-value service, the health system can become more sustainable [3, 10]. There is limited evidence to date on the healthcare utilisation via RPPT across a variety of settings, including emergency and inpatient care [6, 8, 9].

The Western Australia Country Health Service (WACHS) operates one of the largest health services in the world by area, covering 2.55 million square kilometres, across seven regions, with a population of approximately 550,000 [11]. The WACHS Command Centre is an example of RPPT that has been implemented in emergency and admitted health settings across country Western Australia (WA). First piloted in 2012, with an Emergency Telehealth Service, the Command Centre subsequently developed and implemented a range of additional service specialties based on organisational need. Similar to many other telehealth programmes globally, there has been no published evaluation on the implementation of the clinical Command Centre services [12]. Therefore, this study aims to evaluate the implementation and access of rural provider-to-provider telehealth across country Western Australia.

## Methods

### Study design

This research is a retrospective observational analysis based on WACHS Command Centre administrative data for all patient contacts across its five clinical telehealth streams, between 31 August 2012 and 31 December 2023.

### Setting

The WACHS Command Centre is a centralised facility within metropolitan Perth, WA, responsible for the oversight, management, and delivery of technology-enabled emergency and admitted healthcare in country WA. The aim of the Command Centre is two-fold, i) to ensure all country WA communities have equitable access to emergency and admitted healthcare, and ii) support the place-based workforce by providing guidance, advice, and access to specialist knowledge [13].

Five clinical streams have been implemented within the Command Centre between 2012 and 2023: Emergency Telehealth Service (ETS) in 2012; Inpatient Telehealth Service (ITS); and Mental Health Emergency Telehealth Service (MH ETS) in 2018; Midwifery and Obstetrics Emergency Telehealth Service (MOETS); and Palliative Care Afterhours Telehealth Service (PalCATS) in 2022 [13]. These streams can be integrated based on patient need and referring site requirements. The current Command Centre workforce is comprised of a range of clinical positions, including Emergency Medicine Specialists (Fellows of the Australasian College of Emergency Medicine, FACEM), Rural Generalists, Clinical Nurse Consultants, Clinical Nurses, Emergency Nurse Practitioners, Psychiatrists, Midwives, Palliative Care Specialists, and Nurse Educators. These positions are currently filled by 174 salaried staff (80.9 full time equivalent), plus a further 155 contracted medical staff. The staff are based in the centralised facility, as well as across WA, interstate, and internationally. Additionally, there are 15 support personnel responsible for operations and administration.

The place-based provider in country WA initiates a referral to the Command Centre via a centralised number available 24 h a day, seven days a week. A referral is made when deemed necessary by the local provider or site. Smaller sites are often staffed by nurses with varying experience in acute or emergency situations, supported by the local general practitioner. In some cases, there may be no medical cover available. Additionally, no patient is seen by the Command Centre without a referral. The referral is prioritised by a Command Centre Nurse Coordinator and streamed to the most appropriate clinician based on the patient's presenting condition, urgency, and the local provider requirements.

As of 31st December 2023, the Command Centre provides services for 103 WACHS-managed health facilities throughout country WA.^1^ These health facilities are classified into four types, according to their size, capacity, services offered, and workforce capability (Table 1) [11]. Overall, there are approximately 6,000 medical and nursing rural healthcare providers working in these facilities.

**Table 1:** WACHS health facility types

WACHS Health facility type	Number	Description
Regional Resource Centre	6	These form a resource ‘hub’ operating in six of the seven regions, acting as the regional referral centre for diagnostic, acute and surgical services, emergency, inpatient, and outpatient services, and visiting specialist services
Integrated District Hospital	15	These provide diagnostic, emergency, acute, and minor surgical services. Includes low risk obstetrics, aged care, coordination of primary and community-based services
Small Hospital	50	These facilities provide primary care, emergency services, aged care, and minor acute and surgical services
Nursing Post/ Health Centre	32	Delivers basic emergency, primary, and ambulatory care, and public health programs. No inpatient facilities

There is an implementation procedure and checklist for Command Centre services to be enabled in these health facilities. This includes the installation of specific information and communication technology, often set up in the emergency bays, plus site orientation to the services, processes, and service agreements. Not all services are required at all sites, for example the nursing posts do not have inpatient facilities, so would not require Inpatient Telehealth.

### Framework, variables, and sources of measurement

An Implementation Science lens is employed throughout this research. Reach, Effectiveness, Adoption, Implementation, Maintenance (RE-AIM) is a planning and evaluation framework used to assess the impact of an intervention [14, 15]. The Implementation Outcomes Framework (IOF) looks at implementation outcomes of acceptability, adoption, appropriateness, cost, fidelity, feasibility, penetration, and sustainability [16, 17]. There is overlap between the two, with Reilly et al. [18] integrating RE-AIM and IOF by expanding the operational definitions. This is intended to increase the depth of constructs within each framework for additional guidance in planning and evaluation, and suggested for later stage interventions looking to assess and scale-up activities [18]. The expanded RE-AIM and IOF definitions are aligned to research questions and utilised here to guide data analysis and recommendations of areas for future research (see Table 2). This research is focused on the implementation of Command Centre services, so the choice of measurement is focused on the extent of implementation and aims of the service, the where and who, rather than clinical patient outcomes.

**Table 2:** Research questions and associated data measures, guided by the expanded RE-AIM and IOF definitions [18]

RE-AIM dimension	Research Questions	Expanded RE-AIM & IOF definition	Data measure
**Reach** (individual population level)	• What is the reach of Command Centre services throughout country WA, by region and health facility type?	Number of individuals exposed to the intervention	Number and median of patient Command Centre service contacts, by region and health facility type
**Effectiveness** (individual population level)	• Does the Command Centre demonstrate equitable access in terms of age, gender, Aboriginal and Torres Strait Islander status, remoteness, and Socioeconomic status?	The degree to which the intervention is producing its intended effects while assessing potential unintended consequences and changes in quality of life Representativeness of individuals relative to the intended population	Command Centre patient characteristics (age on arrival, gender, Aboriginal and/or Torres Strait Islander status, remoteness index, and SEIFA quintile) (2018–2023) Comparison with country WA emergency presentation data for 2018–2023 [20]
**Adoption** (Organisational level)	• What is the number of sites using Command Centre services?	Number and proportion of settings that participate in or are exposed to the intervention	Number of WACHS sites using Command Centre services and compared to number of WACHS sites not using Command Centre services
	• What is the proportion of patient contacts seen by the Command Centre compared to total emergency department contacts to WACHS sites?	Proportion of the intended settings who deliver or are exposed to the public health intervention	The number of patients contacts seen via Command Centre, as a proportion of total emergency contacts in country WA
	• What are the differences between health facility type and use of Command Centre services?	Proportion of the intended settings who deliver or are exposed to the public health intervention	Case mix of Command Centre service type by health facility type
**Implementation** (Organisational level)	• What is the extent Command Centre services have been implemented across WACHS health facilities?	Consistency of delivery as intended and, in the time, required across staff and organisations	Number of sites enabled with technology for each Command Centre services and number of sites actively using Command Centre services *(Proxy measure)*
**Maintenance** (individual population level)	• What is the reach of Command Centre services by patient contacts over time, from 2012 to 2023, demonstrating maintenance of services?	The extent to which the intervention’s primary outcome is sustained ≥ 6 months	Command Centre patient service contacts by service type and region, over time (2012 to 2023)
**Maintenance** (organisational level)	• What is the adoption of Command Centre services by site over time, from 2012 to 2023, demonstrating maintenance of services?	The public health intervention becomes institutionalised or part of the routine organisational practices and policies	Number of WACHS sites utilising Command Centre services, over time (2012 to 2023)

### Data collection

This study examines WACHS Command Centre clinical service data collected as part of routine healthcare provision, collated into the WACHS Data Warehouse, de-identified, and provided as a curated dataset “Command Centre Data Curated Dataset”. Extracted on 23 April 2024, data were analysed for all five clinical streams between 31 August 2012 and 31 December 2023. Data were analysed in IBM SPSS Statistics software (version 29) and Microsoft Power BI (version 2.122.746.0). The Strengthening the Reporting of Observational Studies in Epidemiology (STROBE) Statement Guideline was employed to guide reporting [19].

### Cohort

Patients were retrospectively identified where treatment included a referral to any of the five Command Centre streams. Each referral made to the Command Centre is termed a “service contact”. The number of referrals during this period determined the sample size.

Utilisation of the Command Centre services are reliant on the place-based provider making a referral to the Command Centre. Data on the individual referring provider is not collected so we are unable to accurately measure the number of individual providers using the service. Therefore, patient service contacts are used as a proxy measure to determine the extent of Command Centre service utilisation at each health facility.

As these services are of an acute or emergency nature (i.e. a one off), no cases were lost to follow-up. Patients may present multiple times across the period.

### Additional data sources

The WACHS “Emergency Collection Data Exploration Tool” [20] was analysed to determine the proportion of patient contacts seen via the Command Centre and effectiveness comparison. Patient residential postcode has been mapped to the Australian Bureau of Statistics Index of Relative Socio-economic Advantage and Disadvantage (IRSAD) number and decile, then converted to quintile [21].

### Bias

The use of administrative health service data leads to selection bias via exposure, where we only have the information of patients who have a perceived need for medical attention, have presented at a WACHS-managed health facility, and where the local provider has subsequently perceived the need to make a referral to the Command Centre. This omits all patients who have made the decision to either not attend a health facility, present at a non-WACHS managed facility, or where the provider has not made a referral to the Command Centre.

### Data analysis

The primary analysis used descriptive statistics, reporting frequency, distributions, and proportions via the RE-AIM domains as described in Table 1. This includes patient characteristics from service data.

#### Missing data

All patient service contacts are present, therefore implementation analyses by patient volume is not affected by missing values. Data are missing not at random (MNAR) between 2012 and 2018 when data systems were being created and established. Main variables not collected during this time are based on patient demographics, affecting 67,121 cases (31.1% of total cases). These are age (55,312, 82.4% missing during this time), sex (55,178, 82.2%), Aboriginal and/or Torres Strait Islander status (56,241, 82.3%), remoteness (55,528, 82.7%), and patient postcode (55,177, 82.2%). The team made the decision to not impute patient demographic data pre-2018, because we would not be able to confirm patient postcode and remoteness accuracy. Therefore, patient characteristic data are presented from 5th January 2018, when new data systems were established, equalling 148,844 cases. Some data are missing at random (MAR) after new systems were established, where variables were either not collected from the patient or not entered into the system by the provider. This data is presented in the results.

#### Age

Age is assessed by patient age on arrival. Age has been grouped for more in-depth analyses, based on alignment to the Australian Bureau of Statistics and international standards [22]. The six broad groups are: under 1, 1–14, 15–24, 25–44, 45–64, and 65 and over.

For the emergency department data, ages > 114 years were removed from the analysis, 22 episodes in total (0.0%). This is based on data from the Australian Institute of Health and Welfare [23], where the maximum age for any one person in Australia has been 114 years.

We conducted a t-test to compare the mean age between the Command Centre and all ED presentations between 2018 and 2023.

#### Remoteness

Remoteness is defined by the Australian Statistical Geography Standard (ASGS) Remoteness Structure into five categories: major cities, inner regional, outer regional, remote, and very remote [24]. This measure is derived from the Accessibility/Remoteness Index of Australia Plus (ARIA +), characterised by a measure of relative geographic access to services and mapped to usual patient residential postcode [25].

#### Acuity (triage category)

The Australasian Triage Scale (ATS) is a clinical tool used in emergency departments to ensure that presenting patients are prioritised according to clinical urgency [26]. There are five categories as follows: 1 – Resuscitation (Immediate), 2 – Emergency (within 10 min), 3 – Urgent (within 30 min), 4 – Semi-urgent (within 60 min), and 5 – Non-urgent (within 120 min).

#### Socio-economic Indexes for Areas (SEIFA)

The Socio-Economic Indexes for Areas (SEIFA), Australia, combines census data including income, education, employment, occupation, housing and family structure to summarise the socio-economic characteristics of an area [21]. The reach of the Command Centre services has been assessed for effectiveness using the Index of Relative Socio-economic Advantage and Disadvantage (IRSAD), a measure of both relative advantage and disadvantage, summarising the economic and social conditions of people and households [21]. This score is grouped into deciles and converted to quintiles (1 to 5) for this analysis, where 1 represents the most disadvantaged population areas, to 5, the more advantaged areas. IRSAD has been mapped to usual patient residential postcode. Additionally, equity measures are best assessed over time to identify and address any inequities when they arise [27]. This data has been presented where available, from 2018 to 2023.

## Results

### Reach

**Fig. 1:**
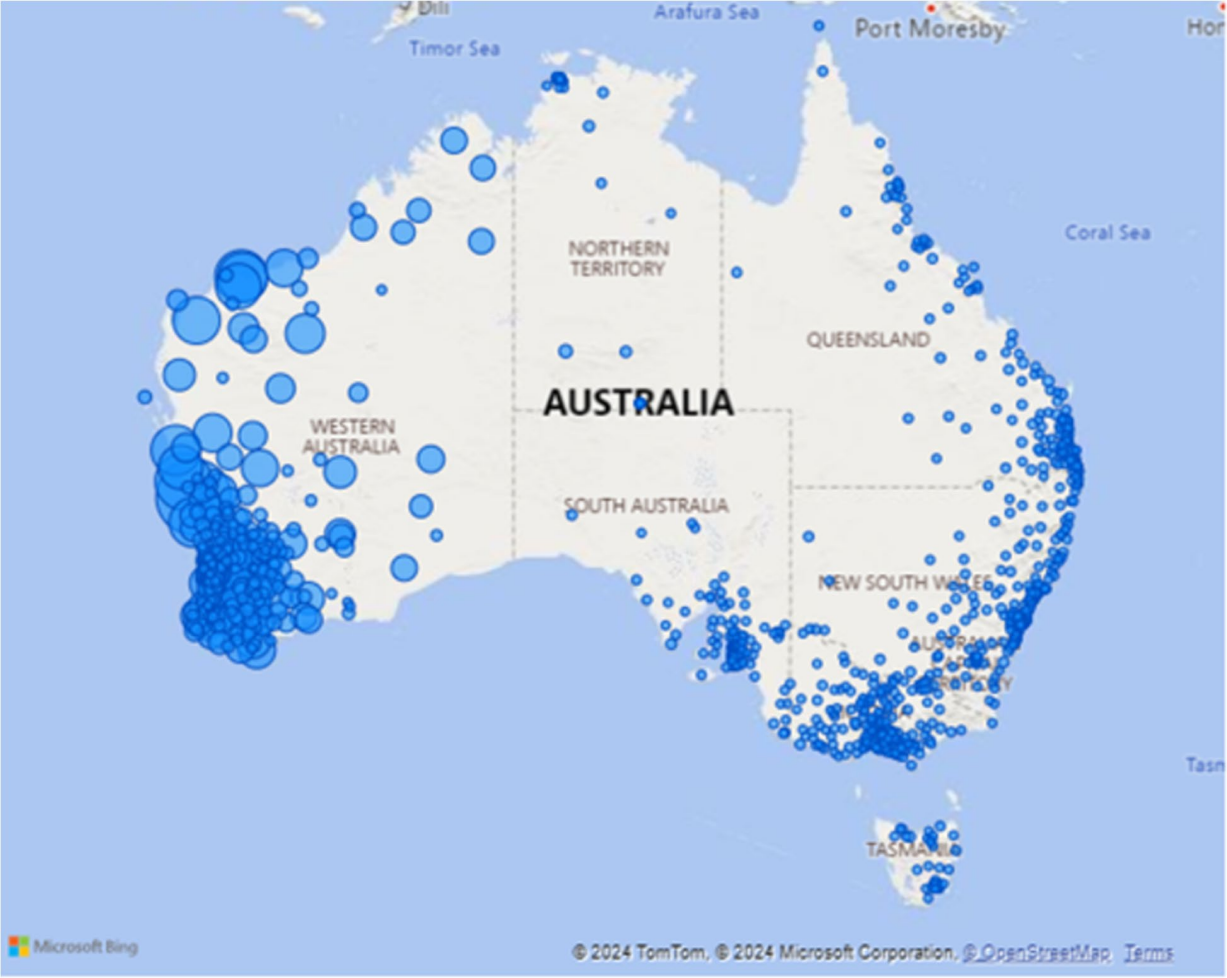
WACHS Command Centre service reach by patient residential postcode, 2018–2023. Size of the blue dot represents number of patient contacts

A total of 215,965 service contacts were analysed from the five Command Centre clinical services between 31st August 2012 to 31 December 2023. The reach of services are illustrated by patient characteristics (Table 3) and usual residential location (postcode) (Fig. 1). The latter shows the location of where patients are from, the majority being from county WA, but shows services delivered to holiday makers or visitors to the regions.

**Table 3:** Patient characteristics for all Command Centre services, by service stream, 2018–2023

Patient characteristics	ETS^a^ N (%)	ITS^a^ N (%)	MHETS^a^ N (%)	MOETS^a,b^ N (%)	PalCATS^a,^^b^ N (%)	All Command Centre services N (%)
**Total**^a^	**133,428 (89.6)**	**2,016 (1.4)**	**9,605 (6.4)**	**3,204 (2.2)**	**591 (0.4)**	**148,844 (100)**
**Age**						
Under 1	1,532 (1.1)	< 5 (0.0)	0 (0.0)	28 (0.7)	0 (0.0)	**1,561 (1.1)**
1–14	23,172 (17.4)	18 (0.9)	499 (5.2)	0 (0.0)	0 (0.0)	**23,689 (15.9)**
15–24	14,249 (10.7)	42 (2.1)	3,035 (31.6)	952 (29.7)	< 5 (0.0)	**18,280 (12.3)**
25–44	29,909 (22.4)	121 (6.0)	4,048 (42.1)	2,174 (67.9)	17 (2.9)	**36.269 (24.4)**
45–64	30,085 (22.5)	269 (13.3)	1,697 (17.7)	< 5 (0.0)	94 (15.9)	**32,147 (21.6)**
65 and over	34,444 (25.8)	1,529 (75.8)	325 (3.4)	0 (0.0)	466 (78.8)	**36,764 (24.5)**
Missing	37 (0.3)	36 (1.8)	1 (0.0)	48 (1.5)	12 (2.0)	**134 (0.1)**
**Sex**						
Female	64,587 (48.4)	996 (49.4)	5,072 (52.8)	3,317 (97.8)	267 (45.2)	**74,059 (49.8)**
Male	68,771 (51.5)	984 (48.8)	4532 (47.2)	19 (0.6)	312 (52.8)	**74,618 (50.1)**
Other	9 (0.0)	0 (0.0)	0 (0.0)	0 (0.0)	0 (0.0)	**9 (0.0)**
Not specified	24 (0.0)	0 (0.0)	0 (0.0)	0 (0.0)	0 (0.0)	**24 (0.0)**
Missing	37 (0.0)	36 (1.8)	< 5 (0.0)	48 (1.5)	12 (2.0)	**134 (0.1)**
**Aboriginal and Torres Strait Islander**						
Yes	26,992 (20.2)	336 (16.7)	3,384 (35.2)	993 (31.0)	118 (20.0)	**31,823 (21.4)**
No	105,219 (78.9)	1,644 (81.5)	6,205 (64.6)	2,162 (67.5)	461 (78.0)	**115,691 (77.7)**
Missing	1,217 (0.9)	36 (1.8)	16 (0.2)	49 (1.5)	12 (2.0)	**1,330 (0.9)**
**Remoteness**						
Major cities	19,942 (14.9)	NA	1,652 (17.2)	123 (3.8)	7 (1.2)	**21,724 (14.8)**
Inner regional	16,452 (12.3)	NA	1,999 (20.8)	556 (17.4)	19 (3.2)	**19,026 (13.0)**
Outer regional	46,883 (35.1)	NA	2,115 (22.0)	1,043 (32.6)	314 (53.1)	**50,355 (34.3)**
Remote	29,051 (21.8)	NA	2,317 (24.1)	862 (26.9)	168 (28.4)	**32,398 (22.1)**
Very remote	16,600 (12.4)	NA	1,127 (11.7)	536 (16.7)	71 (12.0)	**18,334 (12.5)**
Missing	4,500 (3.4)	NA	395 (4.1)	84 (2.6)	12 (2.0)	**4,991 (3.4)**
**IRSAD Quintile**						
Q1 (Most disadvantaged)	25,675 (20.1)	84 (19.5)	2,211 (24.0)	976 (31.5)	180 (31.2)	**29,126 (20.7)**
Q2	55,085 (43.2)	174 (40.5)	2,756 (30.0)	1,008 (32.6)	156 (27.0)	**59,179 (42.0)**
Q3	22,203 (17.4)	69 (16.0)	2,205 (24.0)	625 (20.2)	97 (16.8)	**25,199 (17.9)**
Q4	18,278 (14.3)	69 (16.0)	1,522 (16.5)	406 (13.1)	140 (24.3)	**20,415 (14.5)**
Q5 (Most advantaged)	6,324 (5.0)	34 (7.9)	504 (5.5)	79 (2.6)	< 5 (0.7)	**6,945 (4.9)**

^a^Total results are within row. Service stream and all Command Centre services results are within column. ITS remoteness data not available (NA)

^b^MOETS and PalCATS were active for part of the data collection period

**Fig. 2:**
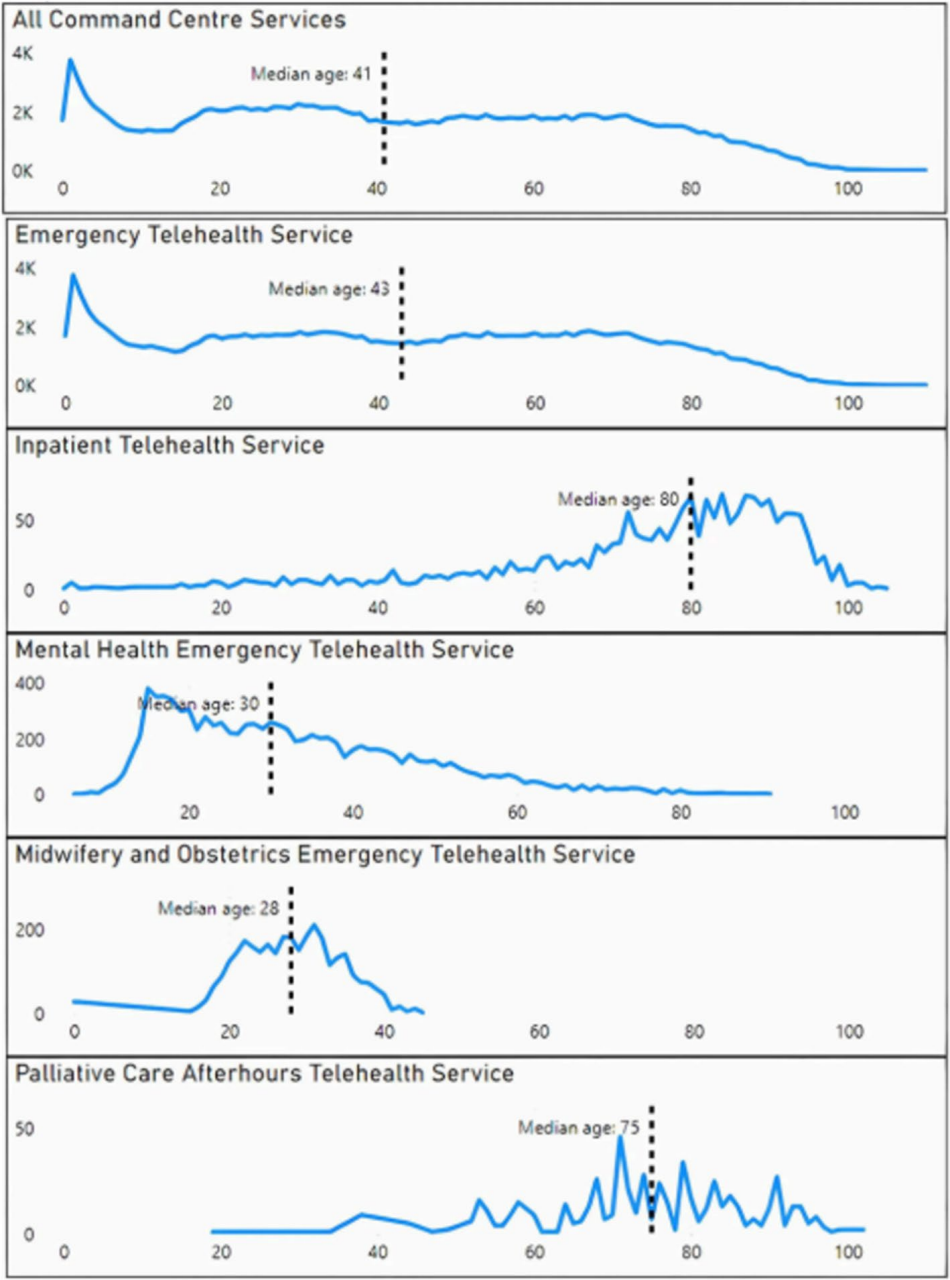
Patient age on arrival for overall and individual Command Centre services, 2018–2023

The highest proportion of patients requiring Command Centre services was in the 65 years and over age bracket. Overall, the median age was 41. When analysed by service type, Inpatient Telehealth Service (ITS) and Palliative Care Afterhours Telehealth Service (PalCATS) showed the highest median patient age on arrival of 80 and 75 respectively, followed by Emergency Telehealth Service (ETS) (43), Mental Health Emergency Telehealth Service (MHETS) (30), and Midwifery and Obstetrics Emergency Telehealth Service (MOETS) (28) (Fig. 2). 49.6% of all contacts were for female patients; MOETS is predominantly female (97.8%) due to the nature of the service. Aboriginal and/or Torres Strait Islander patients represented 21.6% of all Command Centre services contacts.

Reach by WACHS region ranged from 2,652 to 96,539 (Table 4). Highest usage and the greatest number of patient contacts was in the Wheatbelt accounting for 44.7% of all contacts made to the Command Centre, followed by the Midwest (24.9%), Pilbara (13.0%), South West (6.5%), Great Southern (6.2%), Goldfields (3.4%), and Kimberley (1.2%).

**Table 4:** Command Centre service utilisation (reach) by service stream: WACHS region, 2012–2023

		**Command Centre Service Stream**^b^					
**WACHS Region**^a^	**N Health facilities**	**ETS** **N (%)**	**ITS** **N (%)**	**MHETS** **N (%)**	**MOETS** **N (%)**	**PalCATS** **N (%)**	**Total per region** **N (%)**
Goldfields	7	6,142 (82.6)	74 (1.0)	721 (9.7)	307 (4.1)	194 (2.6)	**7,438 (3.4)**
Great Southern	10	12,231 (91.2)	243 (1.8)	419 (3.1)	395 (2.9)	116 (0.9)	**13,404 (6.2)**
Kimberley	13	1,873 (70.6)	10 (0.4)	212 (8.0)	550 (20.7)	7 (0.3)	**2,652 (1.2)**
Midwest	15	51,829 (96.3)	101 (0.2)	1,634 (3.0)	234 (0.4)	49 (0.1)	**53,847 (24.9)**
Pilbara	9	25,028 (89.3)	262 (0.9)	2,212 (7.9)	423 (1.5)	104 (0.4)	**28,029 (13.0)**
South West	13	10,873 (77.4)	350 (2.5)	2,103 (15.0)	704 (5.0)	26 (0.2)	**14,056 (6.5)**
Wheatbelt	28	92,573 (95.9)	976 (1.0)	2,304 (2.4)	591 (0.6)	95 (0.1)	**96,539 (44.7)**
**Total**	**95**	**200,549 (92.9)**	**2,016 (0.9)**	**9,605 (4.4)**	**3,204 (1.5)**	**591 (0.3)**	**215,965 (100)**

^a^Service stream percentages by region are within row. ^b^Total per region are by column

Reach by WACHS health facility type (Table 5) shows the highest usage of Command Centre services was by Small Hospitals, accounting for 81.1% of all contacts. This is followed by Nursing Posts (10.3%), Integrated District Hospitals (7.6%), and Regional Resource Centres (1.0%).

**Table 5:** Command Centre service utilisation (reach) by service stream: WACHS health facility type, 2012–2023

WACHS Health Facility Type^a^	N Health facilities	ETS N (%)^b^	ITS N (%)^b^	MHETS N (%)^b^	MOETS N (%)^b^	PalCATS N (%)^b^	Total per health facility type N (%)
Regional Resource Centre	6	90 (4.3)	< 5 (0.1)	906 (43.7)	854 (41.2)	218 (10.5)	**2,071 (1.0)**
Integrated District Health Service	15	8,820 (53.4)	315 (1.9)	5,373 (32.5)	1,814 (11.0)	193 (1.2)	**16,515 (7.6)**
Small Hospital	50	169,705 (96.9)	1,696 (1.0)	3,017 (1.7)	485 (0.3)	170 (0.1)	**175,073 (81.1)**
Nursing Post / Health facility	22	21,934 (98.3)	< 5 (0.0)	309 (1.4)	51 (0.2)	10 (0.0)	**22,306 (10.3)**
**Total**	**95**	**200,549 (92.9)**	**2,016 (0.9)**	**9,605 (4.4)**	**3,204 (1.5)**	**591 (0.3)**	**215,965 (100)**

^a^Service stream percentages by health facility type are within row. ^b^Total per health facility type are by column

**Fig. 3:**
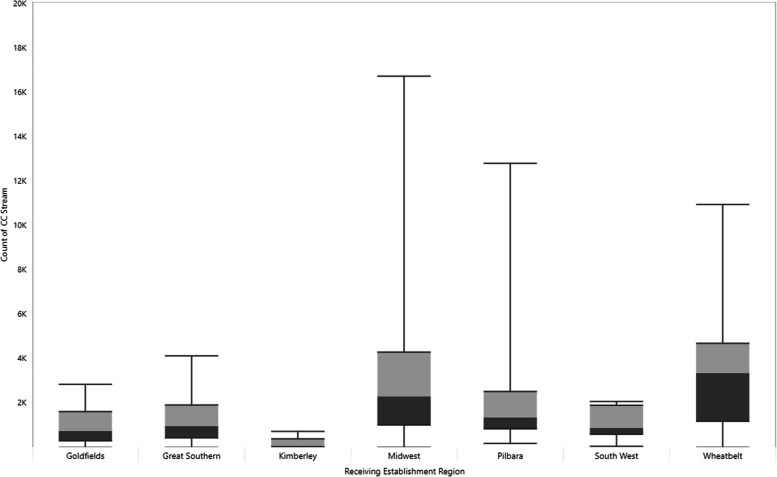
Range of Command Centre service utilisation (reach) by WACHS region, 2012–2023

**Fig. 4:**
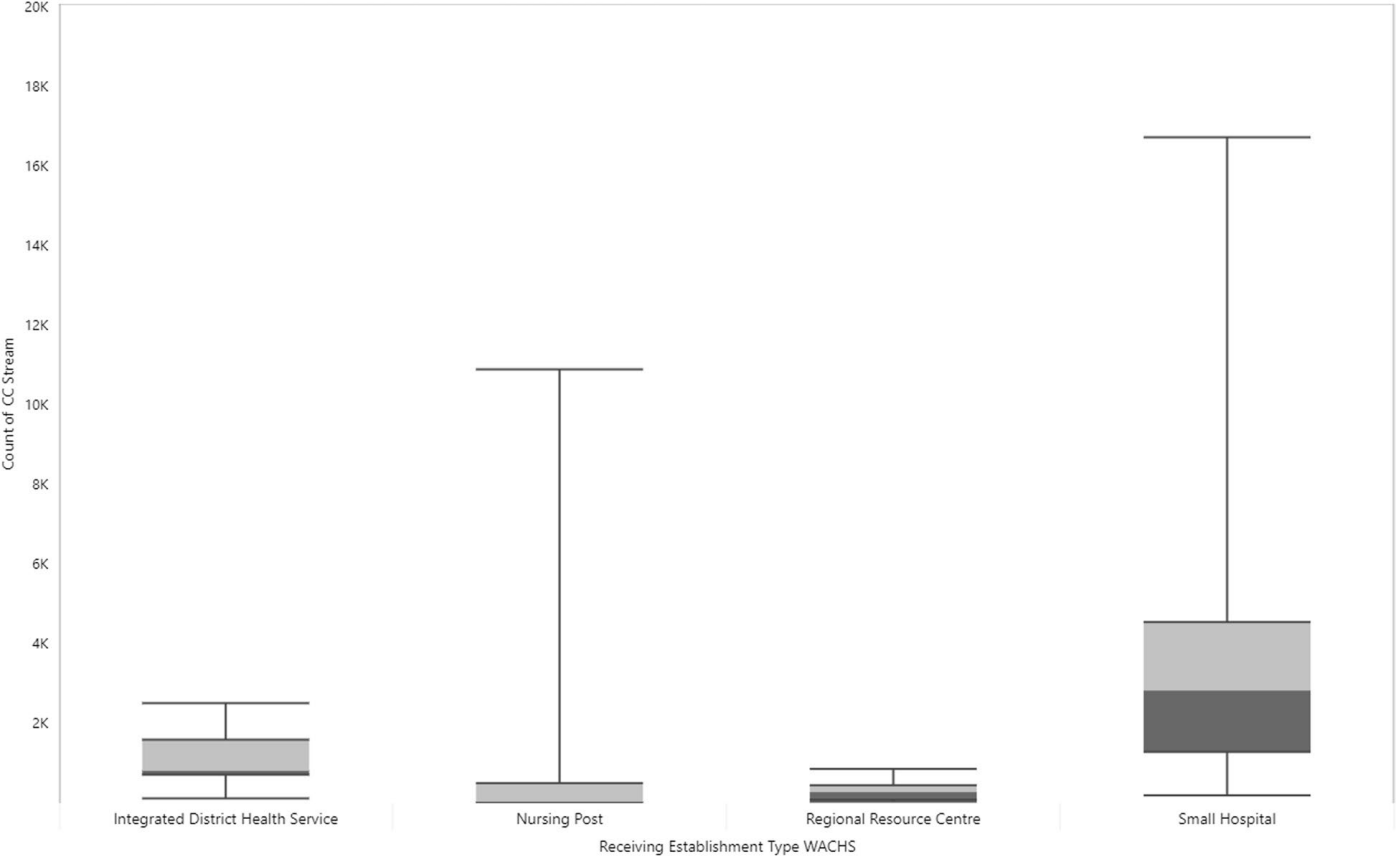
Range of Command Centre service utilisation (reach) by WACHS health facility type, 2012–2023

The distribution of Command Centre usage by region (Fig. 3) and health facility type (Fig. 4) shows a variation in usage between health facilities of between 1 to 16,733 service contacts. Median use is highest in the Wheatbelt (3,357) and Midwest (2,305) regions, with small hospitals demonstrating the highest median for health facility type (2,842.5).

### Effectiveness

**Fig. 5:**
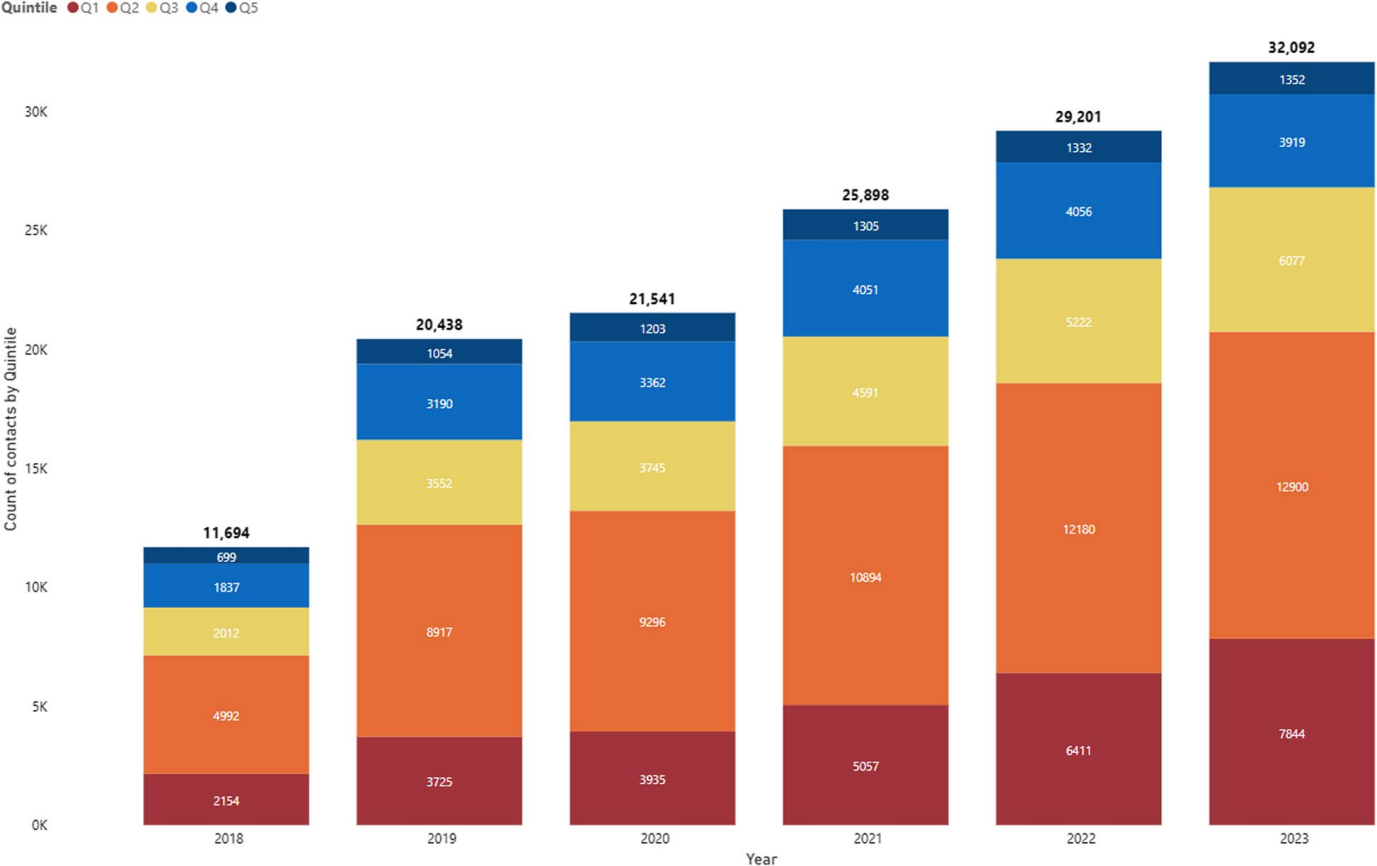
Command Centre service contacts by Index of Relative Socioeconomic Advantage and Disadvantage quintile, 2018–2023

The effectiveness of Command Centre implementation can be assessed by the degree to which the intervention is producing its intended effects and the representativeness of individuals seen relative to the intended population. An increase in utilisation and therefore access to emergency and specialist healthcare where previously not available is seen as a positive outcome. Figure 5 demonstrates an increase in access in all IRSAD Quintiles for the years 2018 to 2023 (where data was available). Of note are referrals to the Command Centre for patients from Q1 (the most disadvantaged) at 20.7% of total referrals and Q2 at 42.0%. In comparison, National ED presentation rates by Q1 and Q2 are 24.2% and 22.9% respectively [28]. The Command Centre services have seen an increase in the proportion of services provided to the most disadvantaged (Q1) from 18.4% in 2018 to 24.4% in 2023, demonstrating Command Centre services are increasingly being delivered to areas of vulnerability.

#### Emergency Department presentations, Country WA

The Command Centre has seen patients with comparable demographic characteristics to ED presentations across country WA of males and females, Aboriginal and/or Torres Strait Islander status, remoteness, and acuity (See Table 6). Of note is the statistically significant (*P* < 0.001) higher mean age of those referred to the Command Centre compared to all ED presentations (42.7 and 38 years respectively). Additionally, the Command Centre has seen a higher proportion of the 65 + age group (24.7% vs 16.9%). Acuity shows The Command Centre is more likely to be contacted in higher acuity cases, with 54.2% of ETS referrals categorised by the Command Centre in triage 1–3, compared to 38.9% of all ED presentations.

**Table 6:** Command Centre service contacts compared to all Emergency Department presentations, country WA, 2018–2023

Measure	Command Centre N (%)	Emergency Department N (%)
Age		
Mean*	42.7	38
Median	41	36
Under 1	1,561 (1.1%)	66,539 (2.5)
1–14	23,689 (15.9)	466,929 (17.7)
15–24	18,280 (12.3)	341,659 (13.0)
25–44	36,269 (24.4)	717,565 (27.2)
45–64	32,147 (21.6)	587,049 (22.3)
65 +	36,764 (24.7)	446,183 (16.9)
Missing	134 (0.1)	8,608 (0.3)
Sex		
Female	74,059 (49.8)	1,296,466 (49.2)
Male	74,618 (50.1)	1,329,066 (50.4)
Other	9 (0.0)	146 (0.0)
Not specified	24 (0.0)	8,866 (0.3)
Missing	134 (0.1)	-
Aboriginal and/or Torres Strait Islander status		
Yes	31,823 (21.4)	654,697 (24.9)
No	115,691 (77.7)	1,951,170 (74.1)
Missing	134 (0.1)	28,677 (1.1)
Remoteness		
Major cities	21,724 (14.8)	319,487 (12.1)
Inner regional	19,026 (13.0)	603,787 (22.9)
Outer regional	50,355 (34.3)	723,461 (27.5)
Remote	32,398 (22.1)	591,409 (22.4)
Very remote	18,334 (12.5)	343,492 (13.0)
Missing	4,991 (3.4)	52,908 (2.0)
Acuity (Triage category)	ETS only	
1—Resuscitation	2,002 (1.1)	12,604 (0.5)
2—Emergency	31,987 (17.7)	269,390 (10.2)
3—Urgent	63,998 (35.4)	742,502 (28.2)
4—Semi-urgent	69,479 (38.4)	1,195,150 (45.4)
5—Non-urgent	13,228 (7.4)	414,886 (15.7)

^*^ t-test *p* < 0.001

### Adoption

A goal of the Command Centre is to support the place-based workforce by providing guidance, advice, and access to specialist knowledge, when required. The Command Centre has a “no wrong door” policy and will assist all referrals when initiated by the place-based provider. We have used patient service contacts as a proxy measure to determine the extent of adoption at each health facility.

**Fig. 6:**
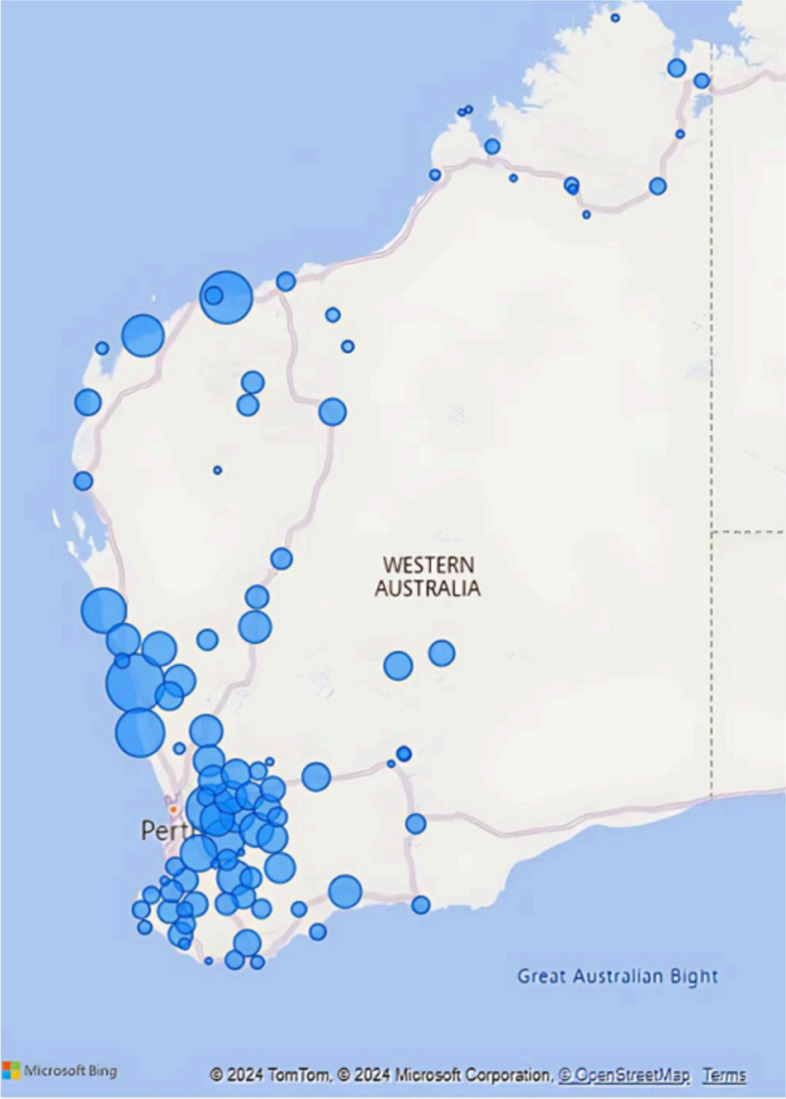
WACHS adoption by health facility location, 2012–2023. Size of circle represents volume of service contacts

**Fig. 7:**
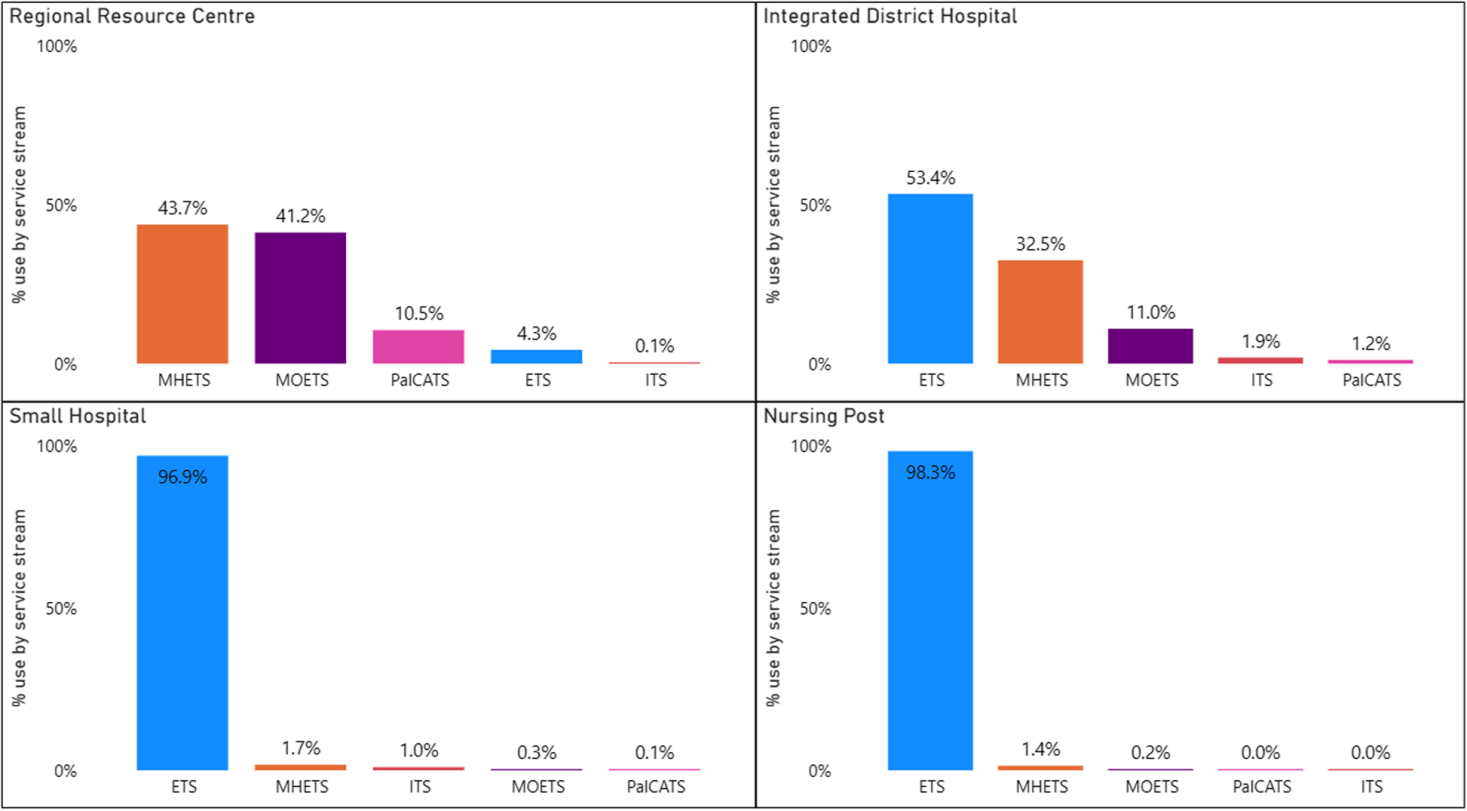
Command Centre reach by “case mix” and health facility type, 2012–2023

As of 31 December 2023, there were 103 WACHS-run health facilities in country WA. Of these, 95 sites (90%) had used Command Centre services (See Fig. 6). The variation between health facility type and use of Command Centre service type are shown in Fig. 7, demonstrating the adoption of each type of service. For example, a Regional Resource Centre is a large facility with broader and larger internal capacity and capability, and the main hub for a region. The adoption of the Command Centre services by these facilities are the lowest, at 1.0% of the total service contacts, likely as they are not required due to sufficient resources at the local site. However, when broken down by service type, it is evident the Regional Resource Centres have adopted MHETS, MOETS, and PalCATS at relatively higher rates, demonstrating the need for more specific service support. In contrast, the Small Hospitals demonstrate a much higher adoption of Command Centre services, at 81.1%, utilising mostly ETS (96.9%). The Integrated District Hospitals and Nursing Posts have adopted services at a relatively similar rate at 7.6% and 10.3% respectively but demonstrate large variability in usage between different regions and individual sites. For example, there are nine nursing posts who have utilised Command Centre services less than ten times across the data reporting period, and one site with over 10,000 contacts.

**Fig. 8:**
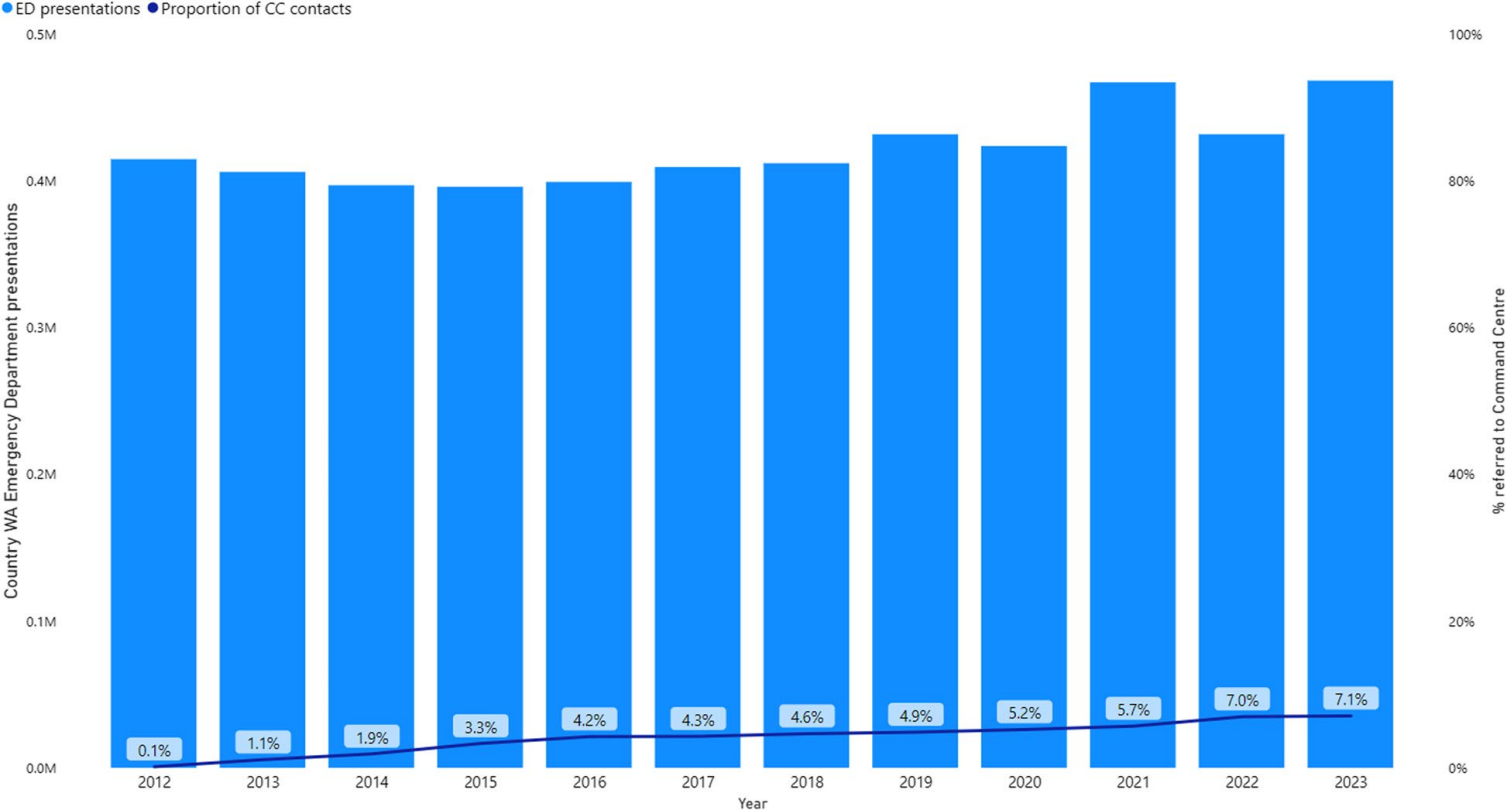
Country WA Emergency Department presentations vs Command Centre contacts, 2012–2023

In the same 12-year period of analysis, 4.3% of all ED presentations in country WA (*n* = 5,056,913) were referred to the Command Centre over this time. This ranges from 0.1% in the first year of implementation, growing to 7.1% in 2023, as demonstrated in Fig. 8.

### Implementation

Early implementation of Command Centre services has been based on organisational need, with a more structured rollout in recent years. There is little information regarding the consistency of delivery as intended and, in the time, required across staff and organisations without additional contextual data. We use a proxy measure to state the current implementation of services throughout country WA.

As of the end of 2023, the technology to fully enable Command Centre services had been fully (all services *N* = 59) or partly (some services *N* = 29) implemented into 88 of the 103 WACHS-managed health facilities throughout country WA. All regional resource centres are partly enabled as they have internal capacity for admitted patients. In total, 95 sites had utilised the services, with seven nursing posts accessing services without being enabled. This is still possible but lacks some of the technological functionality and integration of being enabled. The Command Centre service implementation breakdown is demonstrated in Table 7, including the number of sites who have utilised each service.

**Table 7:** Command Centre service site enablement and utilisation

Command Centre service stream	Number of sites where CC ICT has been implemented	Number of sites utilising the service
Emergency Telehealth Service	85	91
Inpatient Telehealth Service	59	57
Mental Health Emergency Telehealth Service	88	86
Midwifery and Obstetrics Emergency Telehealth Service	87	80
Palliative Care Afterhours Telehealth Service	84	57
**Total**	**88**	**95**

### Maintenance

**Fig. 9:**
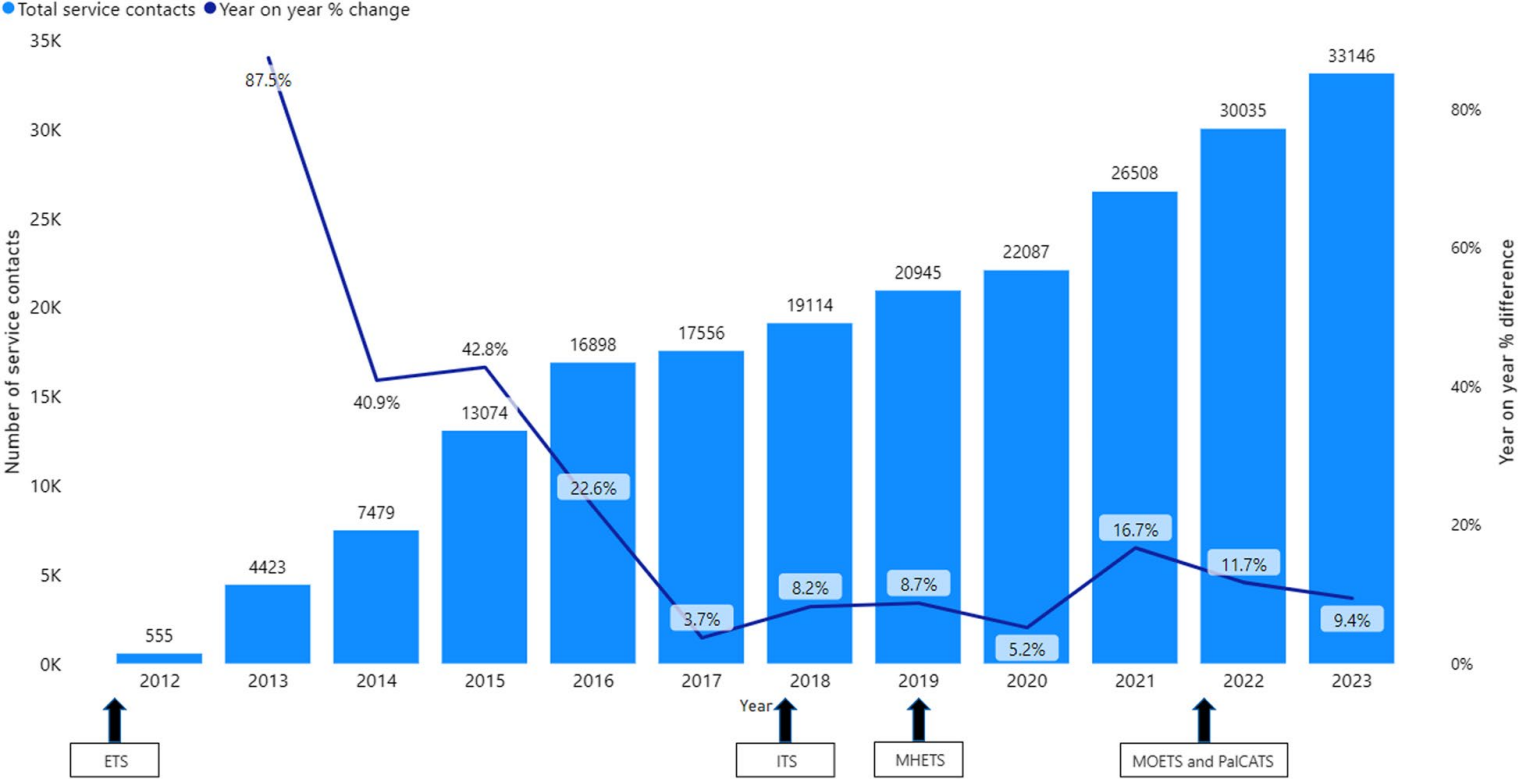
Maintenance of reach and year on year percentage change, 2012 to 2023

Maintenance is defined as the extent to which the intervention’s primary outcome is sustained for more than six months. Results show an increase in reach each year since inception in 2012, with the year-on-year percentage increase shown in Fig. 9. In 2023, this averaged to 90.8 contacts per day, equating to the fourth busiest emergency department in country WA [20].

**Fig. 10:**
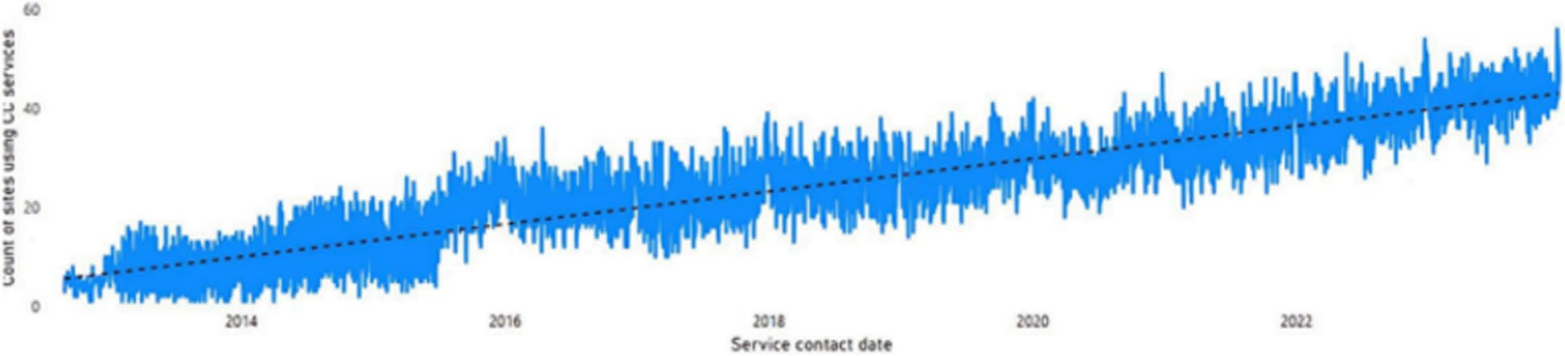
Maintenance in the adoption of individual WACHS sites utilising Command Centre services over time, 2012–2023

Additionally, regarding site-level adoption over time, there is an upwards trend in the number of individual WACHS sites using Command Centre services per day between 2012 to 2023 (Fig. 10). Highest usage was seen on Boxing Day 2023, with 56 different sites utilising Command Centre services. Additional figures of maintenance by service type (Figure S1) and by region (Figure S2) are found in the supplementary file.

## Discussion

Using the expanded RE-AIM and IOF, this observational retrospective study analysed the five RPPT clinical streams from the WACHS Command Centre that have been implemented throughout WACHS-managed hospitals in country WA. Results show there has been a steady increase in both the reach and adoption of these services over time, demonstrating maintenance, but a variation in usage at region, site, and service level. The cause of variation in service usage is largely unknown, with higher usage and variation shown for small hospitals and nursing posts (Fig. 4). These facilities provide crucial services for lower populated areas, but are often limited in terms of staff (capacity, scope), infrastructure, and specialised care. A systematic review in the use of telehealth for non-critical emergencies in rural or remote EDs found usage was increased for facilities with predominantly nursing staff and limited local medical support, similar to the results of this research [29].

Research in Australia and the United States have also shown strong overall growth of RPPT, but variable rates of adoption within rural areas and between urban and rural areas [30–32]. Researchers found the main reasons for a differentiation in uptake were the on-boarding process, clinician resistance, strategic barriers, and the level of telehealth engagement within populations [30, 31]. Additionally, Totten et al. identified several barriers and enablers to the implementation of RPPT, with the two most frequently cited reasons being organisational resources for implementation and access to knowledge and information (staff training and how to incorporate into current workflows) [6]. Many of these barriers and enablers are associated with implementation or change management practices. A systematic scoping review found that the slow adoption of healthcare enabled by technology may be due to a fragmented approach and limited planning of appropriate implementation strategies [33]. Understanding these factors is key to the long-term sustainability of RPPT services and accelerating benefits to both the consumer and provider [34, 35].

Assessing access to a health service is complex and multifaceted, influenced by individual, organisational, and systems level factors. For this research we draw on Levesque's [36] conceptualisation of access, “the opportunity to reach and obtain appropriate health care services in situations of perceived need for care”. This means that, on average, those in equal need of healthcare receive similar treatment, regardless of socioeconomic characteristics [37]. Essentially, those in rural and remote areas should have the same access to emergency and inpatient care compared to those in urban areas. RPPT achieves this by centralising a networked pool of emergency and specialist staff via one access point for all rural and remote health facilities to access when needed. Healthcare enabled by technology can be used as a tool to increase the equity of access to emergency and speciality care, especially for those in rural and remote areas [38, 39].

There are several points in access to healthcare which are assumed for RPPT services. The patient has recognised a need and desire for medical attention and has been able to seek and reach a health facility. Additionally, access to the service is reliant on the local provider to subsequently perceive a need to make a referral to RPPT services [36]. This differs from direct-to-consumer and scheduled healthcare enabled by technology, which is largely reliant on patient initiation and choice, plus provider availability [40]. Therefore, how we measure access and equity may differ, but it remains imperative to do so via good quality monitoring [41].

Given the growing area of research for RPPT and inconsistency measuring equity, there is little to compare the results of this study [6]. One group who has is the Western New South Wales Virtual Rural Generalist Service (VRGS), a similar RPPT model of care in small rural hospitals, designed for lower acuity ED presentations and scheduled inpatient ward rounds [42]. Researchers concluded the VRGS achieved similar outcomes in quality of medical care when compared to non-VRGS care, but also measured access via patient characteristics. VRGS saw patients who were more likely to be younger, less likely to be from a socio-economically disadvantaged group and were classified as lower acuity on presentation when compared to the non-VRGS group [42]. In comparison, the results of this study show patients referred to Command Centre services were significantly older (*p* < 0.001) and classified with higher acuity (54.2% in triage 1–3 vs 47.2%). This suggests the Command Centre has increased access for more emergency and complex cases, and overtime, to those from more disadvantaged areas.

### Limitations

First, this retrospective analysis is constrained by the availability, accuracy, and reliability of data within the administrative data set. Patient characteristic data are missing not at random between 2012 and 2018 when data systems were being established. While the analysis of patient characteristics is limited to the years 2018 to 2023, the primary objective of examining service implementation and patient interactions remains unaffected.

Second, data are unknown before and after the patient service contact. RPPT services in hospital-based emergency and inpatient settings capture one point in time. To capture clinical effectiveness of RPPT interventions and a more complete patient journey, we would require linked data sets and patient-reported outcomes and experience measures.

Third, not all health facilities in country WA are captured. There are additional health facilities such as those managed by Aboriginal Medical Services (AMS) and other organisations. These facilities are not managed by WACHS and therefore out of scope of this research, but present key information especially regarding the equity and representativeness of access to hospital-based emergency and inpatient care in rural and remote WA. Command Centre services have been piloted in one AMS site to assess the acceptability and feasibility of services specifically for Aboriginal and Torres Strait Island populations.

Fourth, there are limitations in assessing the effectiveness of implementation of Command Centre services without additional contextual information from the locations where they have been implemented. The focus of future research should be on the experiences of the end user, the place-based providers and patients accessing RPPT services.

## Conclusion

Rural provider-to-provider telehealth is growing rapidly, delivering greater access to emergency and inpatient healthcare services, and supporting the workforce in rural and remote areas. Here, we demonstrate evidence of service implementation from the WA Country Health Service Command Centre, between 2012 and 2023. The results show a steady increase in both reach and adoption of services across country WA, with wide variation depending on region, site, and health facility type. The results of this study show there is need to further understand the contextual factors and provider perceptions regarding usage of rural provider-to-provider telehealth to demonstrate overall value, tailor adoption strategies, and guide future sustainability and scale-up.

## Supplementary Information

Supplementary Material 1.

## Data Availability

The data that support the findings of this study are available from WA Country Health Service in controlled access data storage. Restrictions apply to the availability of these data due to reasons of sensitivity, which were used under license for the current study, and so are not publicly available.

## Abbreviations

ACEP: American College of Emergency Physicians
AMS: Aboriginal Medical Services
ARIA +: Accessibility/Remoteness Index of Australia Plus
ASGS: Australian Statistical Geography Standard
FACEM: Fellows of the Australasian College of Emergency Medicine
ETS: Emergency Telehealth Service
IOF: Implementation Outcomes Framework
IRSAD: Index of Relative Socio-economic Advantage and Disadvantage
ITS: Inpatient Telehealth Service
MAR: Missing at random
MHETS: Mental Health Emergency Telehealth Service
MNAR: Missing not at random
MOETS: Midwifery and Obstetrics Emergency Telehealth Service
PalCATS: Palliative Care Afterhours Telehealth Service
RE-AIM: Reach, Effectiveness, Adoption, Implementation, and Maintenance
RPPT: Rural provider-to-provider telehealth
SEIFA: Socio-Economic Indexes for Areas
STROBE: Strengthening the Reporting of Observational Studies in Epidemiology
WA: Western Australia
WACHS: WA Country Health Service

## Footnotes

1 There are additional health facilities managed by Aboriginal Medical Services, Aboriginal Community Controlled Organisations, and other organisations. These are out of scope of this research.
