# Supplementary material for: Implementation of rural provider-to-provider telehealth in country Western Australia: a retrospective observational analysis via the RE-AIM framework
Source: BMC Health Serv Res. 2025 Jan 31;25:189. doi: 10.1186/s12913-025-12335-2 (PMC11786510; doi:10.1186/s12913-025-12335-2)
Supplement: Supplementary file 1 — Supplementary Material 1. [file 12913_2025_12335_MOESM1_ESM.docx]

# Supplementary figures


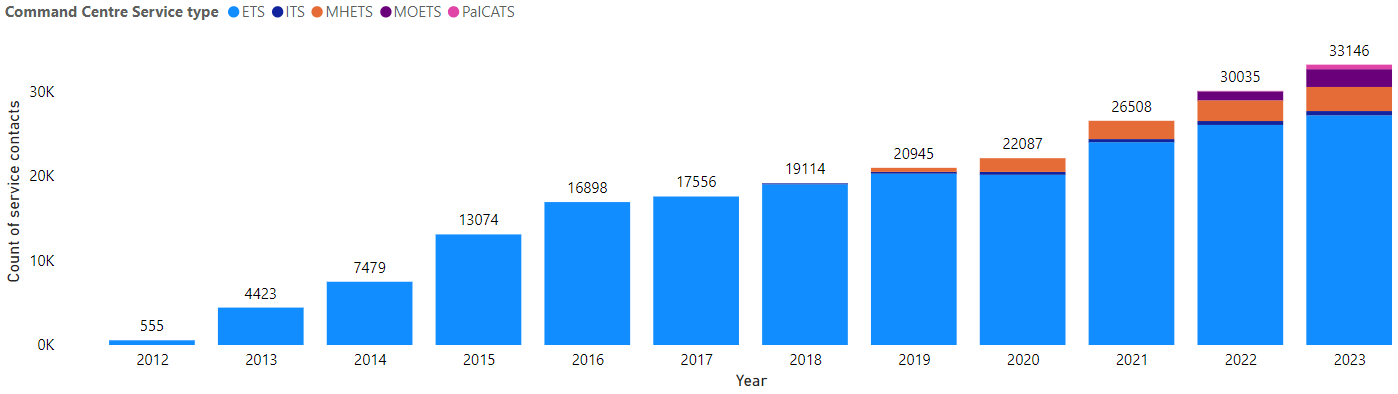


Figure S1. Maintenance in reach of Command Centre services, by service type, over time, 2012-2023.


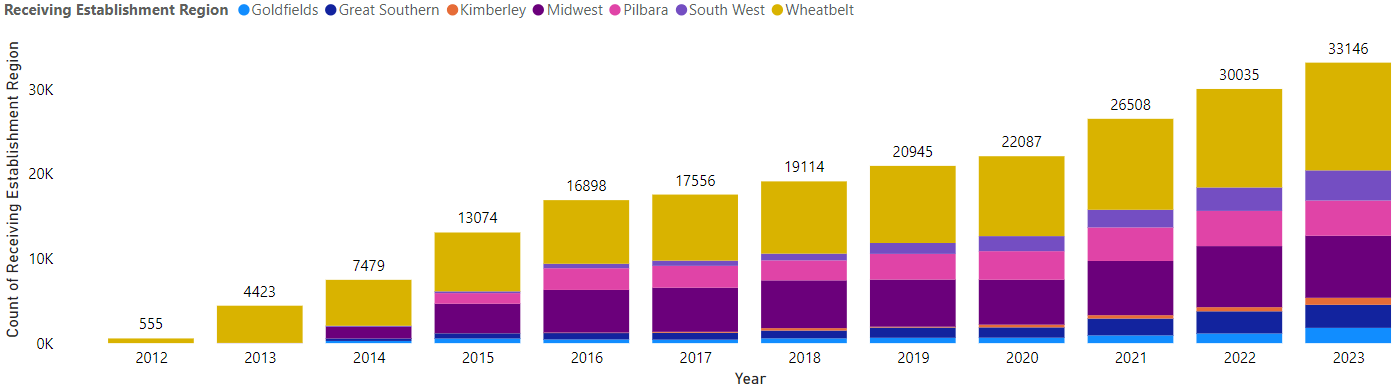


Figure S2. Maintenance in reach of Command Centre services, by region, over time, 2012-2023.
